# A Well Deserved Honor

**Published:** 1887-04

**Authors:** 


### Editorial.

### A Well Deserved Honor.

It is with great pleasure that we make mention of the fact that Dr. Chester H. Harroun, of Toledo, who is well known throughout the profession in this country, has recently had conferred upon him the degree of Doctor of Medicine by a medical college in his own city. This is a well deserved honor, in which all who knew Dr. Harroun will rejoice.

It is all the more gratifying because done by an institution in his own city where his worth and merit are most fully known, and by this act recognized.

A person often feels complimented by honors given to them from a distant point, and by those who are comparatively ignorant of the true character and worth of the recipient.

This honor conferred upon Dr. Harroun may be regarded as one of very high character because based upon thoroughly understood ability and merit.

